# Improved sample treatment protocol for accurate detection of live *Salmonella* spp. in food samples by viability PCR

**DOI:** 10.1371/journal.pone.0189302

**Published:** 2017-12-12

**Authors:** Mai Dinh Thanh, Gemma Agustí, Anneluise Mader, Bernd Appel, Francesc Codony

**Affiliations:** 1 Freie Universität Berlin, Department of Biology, Chemistry and Pharmacy, Berlin, Germany; 2 German Federal Institute for Risk Assessment, Department Biological Safety, Berlin, Germany; 3 GenIUL, Barcelona, Spain; University of Hyogo, JAPAN

## Abstract

Culture-based detection is still considered as the standard way for detection of *Salmonella* in foods, although molecular methods, such as viability PCR (vPCR), have been introduced to overcome some disadvantages of traditional culture methods. Despite the success of the vPCR methodology, the problem of false-positive results is a major drawback, especially when applied to environmental samples, hindering the interpretation of the results. To improve the efficiency of vPCR, many approaches have been introduced by several authors during the last years. In the present work, the combination of PEMAX dye, double tube change, and double photo-activation step was established as a strategy to improve vPCR protocol. By combining these approaches, we developed an improved sample treatment protocol able to neutralize DNA signals of up to 5.0×10^7^ dead cells/sample from both pure culture and artificially contaminated food samples. Our results indicate that vPCR can work reliable and has a potential for high throughput detection of live *Salmonella* cells in food samples, minimizing false-positive signals.

## 1. Introduction

*Salmonella* belongs to one of the most common zoonotic pathogens causing a notable number of foodborne outbreaks and product recalls. In the European Union, 94,625 confirmed salmonellosis cases were reported in 2015 [1]. For the USA, the Centers for Disease Control and Prevention estimate approximately 1.2 million illnesses and 450 deaths every year [2]. The most important food vehicles for foodborne *Salmonella* outbreaks were eggs and egg products, pig meat and products thereof, as well as bakery products [1]. Especially bakery products pose a direct risk to the consumers as no further decontamination steps take place before consumption.

In order to improve food safety related to *Salmonella*, it is important to carry out preventive quality controls based on standard detection technologies. According to several governmental regulations or recommendations, *Salmonella* must not be detectable in 25 g of food. Hitherto, the culture-based detection method is considered as the ideal way for detection of microorganisms. However, this method also shows limits and disadvantages such as skill level required, antimicrobial effects, matrices that inhibit detection completely (e.g. cloves), amount of waste due to duplicates and dilutions, plus it is laborious and time-consuming. For instance, 3 to 4 days are required to obtain a negative result and more than 5 days to confirm a positive one (ISO 6579:2002; [3]). Further, viable but nonculturable (VBNC) cells cannot be detected. An alternative approach is polymerase chain reaction (PCR), a powerful method for the detection of microorganisms in various matrices. PCR is rapid, sensitive and allows specific detection of the target microorganism based on the amplification of their DNA.

The biggest drawback of the classical PCR is that this method cannot differentiate between live and dead populations or extracellular DNA. Viability PCR (vPCR) has been used to selectively detect live microorganisms by molecular procedures. In vPCR, photo-reactive dyes such as ethidium monoazide (EMA), propidium monoazide (PMA) and PEMAX^TM^ (a mix of photo-reactive azide forms of phenanthridium) have been used to exclude DNA from dead cells with compromised cell membrane [4, 5, 6, 7, 8]. By applying a photo-reactive dye to the sample prior to the nucleic acid extraction, the dye can enter compromised cells and can covalently link to the nucleic acid through a photo-activation step, with the result that the amplification of the nucleic acid by molecular tools is inhibited.

However, vPCR seems to have specific advantages and shortcomings. The success of vPCR depends on several factors such as dye (type and concentration) incubation conditions (temperature and time), light source, microorganism and matrices as well as PCR amplification conditions (e.g. temperature and time) used [9–12]. In addition, membrane integrity of the microorganisms affects the vPCR efficiency. Many efforts were made in recent years to optimize the vPCR protocols regarding those critical points. Further, as an approach to solve the problem of “ghost cells”- metabolically inactive and nonculturable cells with intact membrane—Codony et al. [5] combined EMA and PMA to make use of the advantages of both dyes. These authors have shown that the expulsion of EMA (applied in low concentration) from living *Salmonella enteritidis* cells is not a passive process. Hence by adding low concentrations of EMA to PMA in conjunction with vPCR buffers, DNA from “ghost cells” can be bound by EMA, whereas live cells would stay unaffected. Despite many efforts, even using well-optimized vPCR procedures and in spite of the expected full neutralization of nucleic acids of dead microorganisms, it is not unusual to obtain a partial signal reduction. In this sense, recent investigations demonstrated that, at least for *Legionella* and *Salmonella*, a fraction of DNA remains inaccessible to vPCR due to their interaction with the tube wall [6] and in turns generates false positive results.

Based on the cumulated knowledge of the recent years regarding optimization of vPCR protocols, we developed a robust vPCR protocol suitable to exclusively detect live *Salmonella* in several matrices. Our protocol was verified in 33 food samples, which were artificially contaminated with heat killed *Salmonella enterica* cells. Further, it was simulated that food samples are contaminated with *Salmonella* by adding live and dead cells and PCR detection was carried out after enrichment.

## 2. Material and methods

### 2.1. Bacterial strain and culture conditions

*Salmonella enterica* subsp. *enterica* CECT 4594 strain was streaked onto plate count agar (PCA) plates (Liofilchem, Roseto degli Abruzzi, IT) and incubated at 37ºC for 15 to 18 hours. Cells were harvested from the agar plates and suspended in 10 ml phosphate buffered saline (PBS, pH 7.4) to obtain a working bacterial suspension. The cell density was adjusted to an OD_600_ of 0.35, corresponding to 5.0×10^8^ cfu/ml.

### 2.2. Dead cells stock production

To obtain heat killed cells, 1 ml of the cell suspension was heated at 85°C for 35 min using a standard laboratory heat block (thermomixer comfort, Eppendorf, Hamburg, Germany) at 900 rpm. The loss of viability of the cells was verified by plating 100 μl of the cell suspension on PCA plates, followed by incubation for 24 hours at 37ºC.

### 2.3. PEMAX treatment

PEMAX^TM^ dye (GenIUL, Terrassa, Spain), was dissolved in PCR grade water (VWR, Llinas del Vallés, Spain) to obtain a stock dye solution of 2 mM and was kept at -20°C until needed.

All reactions were conducted at a final volume of 200 μl. To create a final concentration of 100 μM PEMAX, 10 μl PEMAX stock solution was added to 200 μl sample. Samples were incubated in the dark at 37ºC for 30 min to allow dye penetration into cells with damaged membranes. Thereafter, the suspension was transferred in a new reaction tube (GenIUL). Photo-induced crosslinking of PEMAX was achieved by exposing the samples to 15 min of light, then 10 min of darkness, followed by 15 min of light using the PhAST Blue instrument (GenIUL) at 100% intensity. Then, the samples were transferred to a new reaction tube and were subsequently centrifuged at 12,100×g for 5 min.

### 2.4. DNA extraction and real-time PCR assay

DNA was extracted using the v-DNA reagent and buffer (GenIUL) according to the manufacturer’s manual. Briefly, the cell pellets were resuspended in 200 μl of v-DNA reagent and were vortexed at 3,200 rpm (MPS-1 Multi Plate Shaker, bioSan, Riga, Latvia) for 5 min. Then, the cells were incubated at 80ºC for 10 min at 1,200 rpm in the heat block. After that, 600 μl of v-DNA buffer were added and vortexed again at 3,200 rpm for 2 min. Thereafter, the samples were centrifuged at 7,500×g for 2 min and 100 μl of supernatant was transferred in a new tube.

Real-time PCR amplifications were performed using PikoReal™ Real-Time PCR System (Thermo Fisher Scientific, Barcelona, Spain) with the following protocol: 15 min at 95°C, 45 cycles of 15 sec at 95°C, 40 sec at 60°C followed by data acquisition. All reactions contained 4 μl of 5X HOT FIREPol Probe qPCR Mix Plus (Solis BioDyne, Tartu, Estonia), 5 μl of DNA template, 0.25 μM of primers and 0.1 μM of specific probe (final volume: 20 μl). The primers and TaqMan probe according to Cheng et al. [13] detected a 262 bp fragment of *Salmonella* spp. Water PCR grade was used as a non-template control.

### 2.5. Amplification efficiency

The real-time PCR efficiency was calculated using the slope of the standard curve, which was generated using 10 fold serial dilutions of *Salmonella* DNA extracted from a pure culture with known concentration. To obtain a standard curve, cycle threshold (C_t_) values were plotted against the corresponding log_10_ cell count. The amplification efficiency (E) was calculated by applying the equation: E = 10−1/slope– 1 [14].

### 2.6. Selectivity of PEMAX on live and dead cells from pure culture

Serial dilutions of live *Salmonella* cells (5.0×10^7^ to 5.0×10^3^ cfu) were mixed with a constant number of 5.0×10^7^ dead *Salmonella* cells and subjected to 0 and 100 μM PEMAX treatment. As controls, samples containing either only 5.0×10^7^ live cells or dead cells were also tested as previously described. The experiments were conducted by two independent assays, each performed in duplicate.

### 2.7. Elimination of dead *Salmonella* PCR signal in food samples

During a 2-month period, a total of 33 food samples and 6 peptone water controls obtained for routine quality controls were subjected to *Salmonella* detection according to the reference culture method (ISO 6579:2002, [3]) and other routine tests (S1 Table) in a local accredited laboratory (Barcelona, Spain). After enrichment, supernatants of the food enrichment broth (FEB) were transported at temperatures between 6-12ºC in a cool box. After arrival at our laboratory, the FEBs were stored at 4°C until usage, which took place within 24–48 h after arrival.

For each sample, 100 μl of the FEB or peptone water control were inoculated with 100 μl of dead *Salmonella* (5.0×10^7^ cells). The mixtures were centrifuged at 100×g for 2 min, in order to remove rough particles. The supernatants were transferred to a new reaction tube and centrifuged again at 12,100×g for 5 min. The cell pellets were suspended in 200 μl of PBS and treated either with 0 and 100 μM PEMAX as described previously. In order to examine the background noise, 100 μl of the FEBs or peptone water controls were mixed with 100 μl of PBS without the addition of dead cells. These mixtures were treated with 0 and 100 μM PEMAX. As a negative and a positive control, 100 μl of dead *Salmonella* cell suspensions mixed with 100 μl of PBS (PBS controls) were subjected to 0 and 100 μM PEMAX as described previously.

### 2.8. Detection of live *Salmonella* in artificially contaminated food samples

Ground pork meat and a ready-to-eat salad mix (*Cichorium intybus* var. foliosum, *Cichorium endivia* var. *latifolium* and *crispum*) obtained from a local supermarket were used for the detection of live *Salmonella* in the presence of dead *Salmonella* cells. For each matrix, 25 g were mixed with 225 ml of peptone water (Oxoid, Basingstoke, UK) and RAPID'Salmonella Capsule (Biorad, Hercules, CA, USA) into a filter stomacher bag (IUL S.A., Barcelona, Spain). Then, 1 ml of live cells (5.0×10^2^ cfu/ml) and 2.5 ml of dead cells (5.0×10^8^ cfu/ml) were inoculated to each suspension and homogenized for 30 sec in a stomacher (Maxicator, IUL, Terrassa, Spain). 250 ml of peptone water inoculated with bacteria were used as control. Contaminated samples were incubated at either 37°C (1 assay with duplicates) or 41.5°C (3 independent assays with duplicates). At point in time 0 and after 24 hours of incubation, 100 μl of supernatants were taken for *Salmonella* detection and were processed as described in 2.7.

## 3. Results

### 3.1. Selectivity of PEMAX on live and dead cells in pure culture

**Fig 1:**
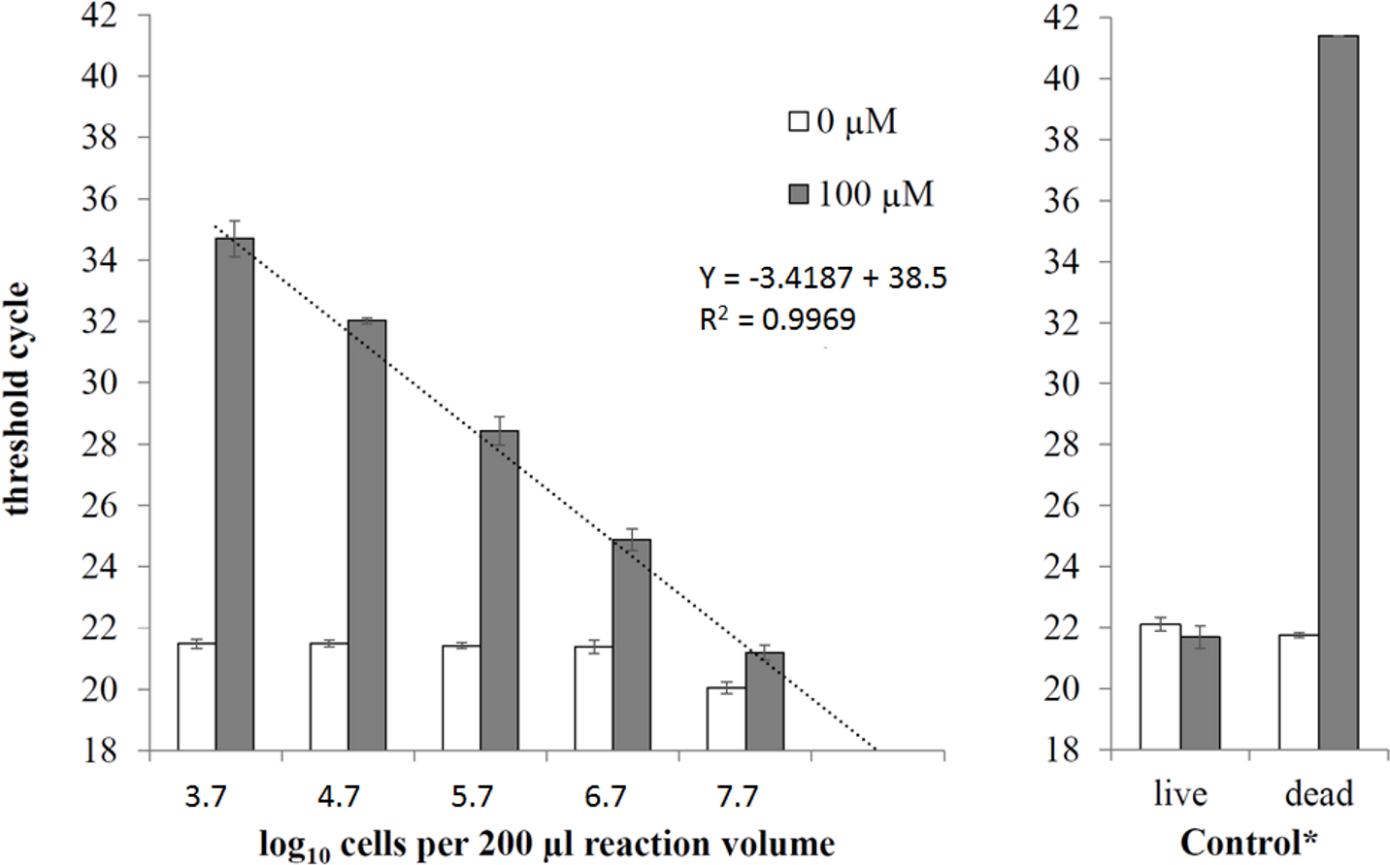
Effect of PEMAX on serial dilutions of live *Salmonella* cells in the presence of dead cells. Serial dilutions of live *Salmonella* cells (5.0×10^7^ to 5.0×10^3^ cfu) in the presence of 5.0×10^7^ dead *Salmonella* cells were treated with 0 μM (white bars) or 100 μM (grey bars) PEMAX followed by real-time PCR. The control samples contains either 5.0×10^7^ live or dead cells (no mixed population). Means, standard deviations (n = 4) and the linear regression equation and R^2^ value of PEMAX treated samples are depicted. * In 3 of 4 dead samples treated with PEMAX no PCR signal were detected, therefore only the PCR signal of 1 sample is indicated in the diagram.

The selectivity of 100 μM PEMAX was tested on serial dilutions of live *Salmonella* cells (5.0×10^7^ to 5.0×10^3^ cfu) mixed with 5.0×10^7^ dead cells. A linear regression analysis was performed by plotting the C_t_ values against the respective log_10_ live *Salmonella* cells (Fig 1, left). Samples without PEMAX treatment showed mean threshold cycles of 21.17 ± 0.63, which were similar to the controls (Fig 1, right) showing mean C_t_ values of 22.11 ± 0.22 for live cells without PEMAX, 21.75 ± 0.36 for dead cells without PEMAX and 21.70 ± 0.09 for live cells with PEMAX.

Samples of live cells treated with PEMAX showed different threshold cycles. Despite the presence of high dead cell concentration, the linear regression curve of PEMAX treated samples did not lose the linearity, showing a correlation coefficient (R^2^) of 0.9969. The PCR efficiency of this experiment was 96.11%. Compared to that, the PCR efficiency with serial dilutions of pure DNA was only slightly higher with 98.27% (S1 Fig). These results indicate that PEMAX was able to eliminate PCR signals of 5.0×10^7^ dead *Salmonella* cells without affecting the PCR efficiency.

As depicted in the linear regression data (Fig 1), the quantification limit of the PCR reaction corresponds to 5.0×10^3^ cells/sample (200 μl). Accordingly, with regard to the DNA extraction and DNA template volume, each PCR reaction contained 31 gene copies. At this gene copy level, the mean C_t_ values were 34.7 ± 0.59. The next tenfold serial dilution step showed no PCR signal. For this reason, C_t_ values between 35 and 39 should be considered below the practical quantification limit. Occasional C_t_ values >39 should be considered as non-significant or a possible fluorescence artifact as results of thermal probe degradation.

### 3.2. Elimination of dead *Salmonella* PCR signal in food samples

FEB of 33 food samples and 6 peptone water negative controls were artificially contaminated with 5.0×10^7^ dead *Salmonella* cells and were subjected to 0 and 100 μM PEMAX treatment. The goal of this experiment was to examine the applicability of vPCR in food samples.

Results are presented in Table 1. The C_t_ values in spiked samples without PEMAX treatment were 23.14 ± 1.16. Once the PEMAX-qPCR procedure has been applied, it was able to completely eliminate the fluorescence signal of 23 samples containing dead *Salmonella* cells; in 2 samples C_t_ values were over 37 and in 8 samples C_t_ values were between 35 and 37. Additionally, samples without the addition of dead *Salmonella* also showed positive PCR signal in 8 cases, although cultural enrichment results showed negative results. As expected, most of the uncontaminated samples with PEMAX treatment showed no PCR signal, however 3 of them showed C_t_ values around 36. Based on these results, C_t_ values over 35 might be background noise and cannot be assessed reliable as positive. By setting the limit of detection to C_t_ 35, all contaminated FEB samples and controls would be assessed as negative.

**Table 1:** Effect of PEMAX on 10^7^ dead *Salmonella* cells in spiked supernatants of the food enrichment broth (FEB).

		vPCR C_t_				Availability in 25 g
		FEB		FEB spiked with 10^7^ dead *Salmonella* cells		25 g food sample
Sample number	Treatment/ Sample name	0 μM PEMAX	100 μM PEMAX	0 μM PEMAX	100 μM PEMAX	RAPID‘ *Salmonella*
1	Curry vegetables	n.s.	n.s.	22.67	n.s.	A
2	Braised beef	n.s.	n.s.	21.79	n.s.	A
3	Spinach with vegetables	n.s.	n.s.	22.68	n.s.	A
4	Meat croquette	n.s.	n.s.	24.90	n.s.	A
5	Potato salad	n.s.	35.77	22.14	n.s.	A
6	Braised beef	n.s.	n.s.	22.36	n.s.	A
7	Vegetable cream	n.s.	n.s.	21.70	n.s	A
8	Beans	36.53	n.s.	21.14	n.s.	A
9	Broad beans	37.83	36.38	22.91	n.s	A
10	Omelet	37.7	n.s.	22.65	n.s.	A
11	Green salad, local market 1	n.s.	n.s.	24.82	n.s.	A
12	Green salad, local market 2	n.s.	n.s.	25.55	35.47	A
13	Green salad, local market 3	35.62	n.s.	22.18	35.92	A
14	Green salad, local market 4	n.s.	n.s.	22.75	35.52	A
15	Green salad, local market 5	n.s.	n.s.	22.21	n.s.	A
16	Vegetable soup	n.s.	36.23	23.18	n.s.	A
17	Grilled fish	n.s.	n.s	23.04	n.s.	A
18	Curry vegetables	n.s.	n.s.	22.12	38.54	A
19	Grilled sausage	37.51	n.s.	24.41	n.s	A
20	Seafood salad	n.s.	n.s.	24.76	35.62	A
21	Sausage with vegetables	n.s.	n.s.	24.22	n.s.	A
22	Stewed lentils	n.s.	n.s.	22.93	n.s.	A
23	French fries	n.s.	n.s.	24.60	36.93	A
24	Macaroni	31.01	n.s	22.32	35.75	A
25	Fish soup	n.s.	n.s.	22.86	n.s.	A
26	Fried fish	n.s.	n.s.	23.16	n.s.	A
27	Lasagne	n.s.	n.s.	21.54	n.s.	A
28	Vegetable soup	34.47	n.s.	21.73	n.s.	A
29	Burger	n.s.	n.s.	23.13	n.s.	A
30	Curry vegetables	n.s.	n.s.	24.30	38.36	A
31	Chicken in sauce	n.s	n.s.	24.44	n.s	A
32	Mashed potatoes	n.s.	n.s.	24.08	35.25	A
33	Grilled chicken	n.s.	n.s	24.35	35.17	A

Artificially contaminated supernatants of the food enrichment broth (FEB) (10^7^ dead *Salmonella* cells per sample) and uncontaminated FEB were treated with 0 μM and 100 μM PEMAX, followed by real-time PCR. C_t_ values are indicated. Further, cultural routine test results for the respective food sample are shown..s.: no signal; A: Absence in 25 g RAPID’ *Salmonella* Medium (Bio-Rad, Hercules, USA)

### 3.3. Detection of live *Salmonella* in artificially contaminated food samples

Salad and ground pork meat were artificially contaminated with 20 cfu/g live and 5×10^7^ cfu/g dead *Salmonella* cells, respectively. Enrichment was conducted at 37°C and 41.5°C for 24 hours in peptone water. The aim of this study was to demonstrate that PEMAX treatment can fully suppress PCR signals of dead cells in food samples without showing any negative effect on the detection on live cells after enrichment.

The results are summarized in Table 2. At point in time 0 hours, the mean C_t_ values of all samples without PEMAX treatment were 30.34 ± 1.29, whereas PEMAX treated samples showed negative PCR results. After 24 hours of enrichment at 37°C, untreated and PEMAX treated samples showed comparable results with mean C_t_ of 29.05 ± 2.13 and 29.64 ± 2.26, respectively, showing the amplification signal of live cells growing in the samples. However, enrichment at 41.5°C could yield mean C_t_ of 24.05 ± 3.53 and 24.78 ± 3.77 for untreated and PEMAX treated samples, respectively, showing that at 41.5ºC, as expected, the cells had grown more efficiently and faster than at 37ºC. In addition, the C_t_ values for the particular food samples varied heavily, resulting in the high standard deviation of the given C_t_ values. For both enrichment temperatures, salad showed higher C_t_ values than meat or the control.

**Table 2:** Detection of live *Salmonella* in artificially contaminated food samples (with live and dead cells) by vPCR method.

	Time	0h		24h	
	Treatment	0 μM PEMAX	100 μM PEMAX	0 μM PEMAX	100 μM PEMAX
Temperature	Sample				
37°C (n = 2)	Control	30.39 ± 0.64	>40*	26.61 ± 0.29	27.23 ± 0.25
	Meat	30.29 ± 0.11	>40*	29.21 ± 0.04	29.44 ± 0.17
	Salad	32.38 ± 0.37	>40*	31.35 ± 0.2	32.26 ± 0.29
41.5°C (n = 6)	Control	30.44 ± 1.55	>40**	22.97 ± 0.55	23.52 ± 0.53
	Meat	29.99 ± 1.14	>40**	22.01 ± 2.78	22.49 ± 3.09
	Salad	29.93 ± 1.35	n.s.	27.19 ± 4.03	28.35 ± 3.88

Threshold cycle (C_t_) value of meat and salad artificially contaminated with *Salmonella* before and after enrichment. Enrichment was conducted at 37°C and 41.5°C. Samples were either let untreated (0 μM PEMAX) or were treated with 100 μM PEMAX. n.s. no PCR signal in 6 samples

* 1 sample out of 2 without PCR signal

** at least 4 samples out of 6 without PCR signal

## 4. Discussion and conclusion

### 4.1. vPCR protocol improvement

In theory, vPCR is a rapid, sensitive, and reliable method to simply detect live cells in a sample by molecular methods. However, the practical experience from many researchers raised doubts about their effectiveness [12, 15, 16]. Despite their huge potential, incomplete suppression of PCR signals resulting in false positive results, in particular with high background of dead cells [17–19], has been one of the major limitations in the spread of this technique and their application in routine quality control. To address this issue, researchers around the world have made efforts for improving the effectiveness of vPCR methodology in order to detect only live cells in the sample. Factors such as the ratio between viable and dead bacterial cells, organic material composition and concentration have been highlighted as potential inhibitors for the vPCR procedure, DNA extraction and qPCR [11, 15].

With the aim to overcome false-positive results, we developed an improved vPCR protocol. In the present work, the improved vPCR protocol was tested in various experiments to demonstrate the applicability of this in real food samples. In these studies, we combined several improvements suggested in the literature in conjunction with our previous vPCR works regarding *Salmonella* in pure cultures [6]. Our focus was to keep the protocol as simple as possible and suitable for adaption in the routine quality control.

In most of the vPCR experiments, heat treatment was used to obtain dead bacteria cells [4, 17–19], therefore we also chose this method to inactivate the cells. Nevertheless, thermally-treated bacteria might differ in their performance compared to bacteria treated with other inactivation processes.

Our killing condition could successfully kill the cells without releasing DNA as the signal of live and dead cells were similar (Fig 1). In the case of heat treatment, cell membrane becomes compromised and viability dyes can easily penetrate into the cell. In our case, we chose the newly appeared reagent PEMAX, which is a blend of viability dyes with different molecular weights. One of them has a certain level of permeability in intact cellular membranes. At least for *Salmonella*, previous investigations have demonstrated that metabolically active cells can be able to extrude certain levels of viability dyes (EMA) that crossed the cellular membrane. By keeping the less selective dye in a smaller concentration, e.g. 10 μM EMA and 50 μM PMA, live cells were not affected [5]. Indeed, in case of our *Salmonella* vPCR experiment, no difference occurred between PEMAX treated and untreated live cells. By using PEMAX, we extended the possibility to avoid detection of “ghost cells”, which have an intact membrane but are metabolically inactive. Since, the *Salmonella* detection is done after the enrichment to ensure the absence of Salmonella in 25 g, the use of the viability dye PEMAX ensures the detection of cells with intact cell membrane and active metabolism.

With our improved vPCR protocol, PCR signal of dead cells with concentrations of up to 5×10^7^ cells/sample could be completely suppressed. Complete signal reduction for *Salmonella* spp. has been obtained before by Xiao et al. [19] by using PMA, but only for concentration up to 10^6^ cell/ml. In their experiments, PMA concentration of up to 200 μM was applied, yet DNA of dead cells with concentrations beyond 10^6^ cells/ml could not be neutralized. On the other hand, Martin et al. [17] were able to neutralize huge amounts of approximately 10^8^ dead cells/g by applying 100 μM PMA and using large amplicons as a PCR target. Nonetheless, in this case the method for preparing dead cells was highly destructive (boiling for 10 min) for the membranes. Treatment with high temperature around 100°C could lead to a release of DNA or make the membrane highly accessible to viability dyes [20–22].

For improving the effectiveness of vPCR, we made use of the findings of Li and Chen [23] as well as Martin et al. [17]. In their studies, the authors tested different primers resulting in PCR amplicon lengths varying between 65 and 260 or 75 and 417 bp, respectively. Their results showed that amplifications of PMA treated samples using primers for long amplicons could yield higher PCR signal suppression than short amplicons. Further, we adopted the tube change approach published by Agustí et al. [6] to our protocol and extended it for a further tube change step after the photo-activation. By changing the tubes after the dark incubation these authors could yield a further signal suppression of 5 C_t_ compared to the control group. Further they showed that by changing the tube, residual DNA attached to the reaction tube could be avoided.

Moreover, the samples were treated with a double photo-activation step of dye, however without additional reagent addition. To our knowledge, this procedure has not been previously reported. By applying double photo-activation with a short dark period in between, we could extent the signal suppression for dead cells by at least 2 C_t_ (S2 Fig). Thus, this simple approach provides a new way to improve the effectiveness of vPCR. Some authors reported the use of double dye treatment, which increased the ΔC_t_ of dead cell samples around 1–2.8 C_t_ compared to a single dye treatment for *Listeria monocytogenes* and *Mycobacterium avium* subsp. *paratuberculosis* [24, 25]. Nevertheless, complete suppression of the fluorescent signals could not be obtained. In our study, a comparable trend was obtained by applying only one dye dosage coupled with double photo-activation instead of double dye treatment.

Our results showed that the ratio of live to dead cells did not affect the efficiency of the method (Fig 1) as some reports indicate [21, 26]. These reports showed that the quantification of live cells by vPCR in the presence of high numbers of dead cells was difficult when the concentration of live cells was lower than 10^5^ cells per sample.

Not least, dye incubation time and temperature is also a critical point affecting the effectiveness of vPCR. Nkuipou-Kenfack et al. [18] reported a combined effect of these two factors; for *Salmonella* an increased in ΔC_t_ of about 2 for incubation at 40°C instead of 20°C could be shown. A further increase of about 2 C_t_ could be yielded when dye incubation time was 30 min instead of 5 min. Based on this finding, we set the PEMAX incubation condition to 30 min at 37°C to improve the vPCR protocol.

### 4.2. Detection of *Salmonella* in food samples

Usually, real samples are complex matrices, which may interfere with the efficacy of the vPCR treatment [10], therefore the new protocol was evaluated for dead *Salmonella* cells in various food samples. With our vPCR protocol using PEMAX, we could yield complete PCR signal suppression for 5.0×10^7^ dead *Salmonella* cells per reaction in most of FEB samples (23 out of 33 samples). Nevertheless, we still have positive signals with C_t_ beyond our quantification limit in 10 samples. Within our practical quantification limit of 35 C_t_, PCR signals below the limit could reliably be assessed as positive. The theoretical detection limit was about 38 C_t_, but signals higher than 35 C_t_ might be caused by residual unspecific amplification or due to thermal degradation of the Taqman probe.

In addition, C_t_ values beyond 35 could not be assessed as reliable, because corresponding gene copy number per PCR reaction was low and would also cause false negative results. As the results for cultural detection were negative for the 10 mentioned FEBs, the positive PCR signals over C_t_ 35 did now mean the presence of *Salmonella*. On the other hand, no signal would not mean negative result. In case that vPCR should become a routine test for *Salmonella*, there is a need to find a common sense for setting the practical quantification limit. Additionally, it should be discussed about further procedures to approve the results beyond the real-time PCR quantification limit. In our opinion, it is not necessary to choose between vPCR and cultural detection method. To the contrary, a combination of both methods would be an ideal way to detect live and viable but nonculturable cells.

Since food matrices can be very complex, maybe some positive signals beyond or around C_t_ 35 could also be explained by incomplete DNA neutralization as result of matrix interference. In order to overcome this problem, the use of detergents during the dye incubation [11] might contribute to an improvement. However, we did not explore this approach because under our point of view, *Salmonella* detection in routine analysis is usually done after 24 hours of enrichment, as regulations claim the absence of live cells in 25g. In case that the samples contain live *Salmonella* cells, C_t_ values would reach the reliable range for positive signals after enrichment due to growth of cells. This criterion is well supported by the results shown in Table 2. In this sense, practical quantification limit of 35 C_t_ (corresponds to 5.0×10^3^ cells/sample) does not meet the requirement for quantification of low concentration, but is sufficient for qualitative detection.

In the last experiment, we demonstrated the applicability of the improved vPCR protocol to a more realistic scenario. Since our aim was to mimic a real world scenario, we decided for lower concentration of dead cells (5.0×10^7^ cells/g = 5.0×10^5^ cells/sample) than tested in the experiment before (10^7^ cells/sample). The start inoculum of live cells was 20 cfu/g (accordingly, 0.2 cfu/sample). This concentration was considered to be sufficient to demonstrate the ability to detect low concentrations after enrichment and still ensure the presence of live cells in the contaminated food sample. The results indicate that the improved vPCR protocol was able to selectively detect live cells without being affected by dead cells. After enrichment, a clear positive signal could be observed, however C_t_ values varied. The reason might be the presence of other microorganisms in meat and salad. The autochthonous flora of salad might cause a competitive situation in the sample during enrichment, which affect the growth of Salmonella. In addition, meat is a further source of nutrition and might affect the growth of *Salmonella* positively.

### 4.3. Summary and conclusion

The present report showed that with the use of combined procedures the results obtained with vPCR are improved and the critical weaknesses of this technique might be solved. These minimal changes in the classical vPCR procedure provides hope to work with complex samples getting complete PCR signal reduction in dead cells avoiding thus, the false positive results. In the present work, a serial dilution of live cells could be detected properly without losing the linearity despite the presence of high dead cell concentration. Our improved vPCR protocol could demonstrate that the problem of false-positive results can be overcome and the same approach can probably be effective for detection of other food pathogens as well.

In conclusion, our proposed vPCR protocol using PEMAX might be suitable for usage in routine analysis, because it is simple and showed reliable suppression of dead cells. In addition, this protocol harbors a potential for high throughput detection because it is quick and easy to conduct.

## Supporting information

S1 Table Microbiological status of 33 food samples tested in a laboratory accredited according to ISO 9001:2008. (DOCX)

S1 Fig *Salmonella* standard curve. Cycle threshold (C_t_) values were plotted against the corresponding log_10_ cell count (3.7–8.7).
(TIF)

S2 Fig Comparison of single and double light treatment. (TIF)
