# Supplementary material for: Improved sample treatment protocol for accurate detection of live Salmonella spp. in food samples by viability PCR
Source: PLoS One. 2017 Dec 12;12(12):e0189302. doi: 10.1371/journal.pone.0189302 (PMC5726647; doi:10.1371/journal.pone.0189302)
Supplement: S1 Table — (DOCX) [file pone.0189302.s001.docx]

Supplement

Microbiological status of 33 food samples tested in a laboratory accredited according to ISO 9001:2008.

|  | Microorganism | Total aerobic mesophilic bacteria | *Entero-bacteriaceae* | *Staphylococcus aureus* | *Salmonella* spp. | *Escherichia  coli* | *Listeria monocytogenes* |
| --- | --- | --- | --- | --- | --- | --- | --- |
|  |  | cfu/g | cfu/g | cfu/g | availability in 25 g | cfu/g | cfu/g |
|  | Method | ISO 4833- 1:2013 | ISO 21528-2:2004 | ISO 6888- 1:1999 | RAPID‘ *Salmonella* | ISO 16649-2:2001 | ISO 11290-2:1998 |
| Sample number | Treatment/  Sample name |  |  |  |  |  |  |
| 1 | Curry vegetables | <10 | <10 | <10 | A | <10 | <10 |
| 2 | Braised beef | <10 | <10 | <10 | A | <10 | <10 |
| 3 | Spinach with vegetables | <10 | <10 | <10 | A | <10 | <10 |
| 4 | Meat Croquette | 180 | <10 | <10 | A | <10 | <10 |
| 5 | Potato salad | >300,000 | 24,000 | <10 | A | 150 | <10 |
| 6 | Braised beef | <10 | <10 | <10 | A | <10 | <10 |
| 7 | Vegetable cream | <10 | <10 | <10 | A | <10 | <10 |
| 8 | Beans | <10 | <10 | <10 | A | <10 | <10 |
| 9 | Broad beans | 300 | <10 | 50 | A | <10 | <10 |
| 10 | Omelet | 40 | <10 | 30 | A | <10 | <10 |
| 11 | Green salad, local market 1 | >300,000 | >30,000 | <10 | A | 10 | - |
| 12 | Green salad, local market 2 | >300,000 | 30,000 | 10 | A | 10 | - |
| 13 | Green salad, local market 3 | 2,00,000 | 139,000 | <10 | A | <10 | - |
| 14 | Green salad, local market 4 | 110,000 | 44,000 | 10 | A | <10 | - |
| 15 | Green salad, local market 5 | >300,000 | 18,000 | <10 | A | <10 | - |
| 16 | Vegetable soup | 11,000 | <10 | <10 | A | <10 | <10 |
| 17 | Grilled fish | <10 | <10 | <10 | A | <10 | <10 |
| 18 | Curry vegetables | <10 | <10 | <10 | A | <10 | <10 |
| 19 | Grilled sausage | 130 | <10 | <10 | A | <10 | <10 |
| 20 | Seafood salad | 200,000 | <10 | <10 | A | <10 | <10 |
| 21 | Sausage with vegetables | 30 | <10 | <10 | A | <10 | <10 |
| 22 | Stewed lentils | <10 | <10 | <10 | A | <10 | <10 |
| 23 | French fries | 10 | <10 | <10 | A | <10 | <10 |
| 24 | Macaroni | 20 | <10 | <10 | A | <10 | <10 |
| 25 | Fish soup | 200 | <10 | <10 | A | <10 | <10 |
| 26 | Fried fish | <10 | <10 | <10 | A | <10 | <10 |
| 27 | Lasagna | 430 | <10 | <10 | A | <10 | <10 |
| 28 | Vegetable soup | <10 | <10 | <10 | A | <10 | <10 |
| 29 | Burger | 110 | <10 | <10 | A | <10 | <10 |
| 30 | Curry vegetables | 10 | <10 | <10 | A | <10 | <10 |
| 31 | Chicken in sauce | <10 | <10 | <10 | A | <10 | <10 |
| 32 | Mashed potatoes | 42 | <10 | <10 | A | <10 | <10 |
| 33 | Grilled Chicken | <10 | <10 | <10 | A | <10 | <10 |

A: Absence in 25 g; - not determined
ISO 4833-1:2013. Microbiology of the food chain -- Horizontal method for the enumeration of microorganisms -- Part 1: Colony count at 30 degrees C by the pour plate technique.
ISO 21528-2:2004. Microbiology of food and animal feeding stuffs -- Horizontal methods for the detection and enumeration of Enterobacteriaceae -- Part 2: Colony-count method.
ISO 6888-1:1999. Microbiology of food and animal feeding stuffs -- Horizontal method for the enumeration of coagulase-positive staphylococci (Staphylococcus aureus and other species) -- Part 1: Technique using Baird-Parker agar medium.
ISO 16649-2:2001. Microbiology of food and animal feeding stuffs -- Horizontal method for the enumeration of beta-glucuronidase-positive Escherichia coli -- Part 2: Colony-count technique at 44 degrees C using 5-bromo-4-chloro-3-indolyl beta-D-glucuronide.
ISO 11290-2:1998. Microbiology of food and animal feeding stuffs -- Horizontal method for the detection and enumeration of Listeria monocytogenes -- Part 2: Enumeration method.
RAPID’ *Salmonella* Medium (Bio-Rad, Hercules, USA)
