# Supplementary figures and images for: Improved sample treatment protocol for accurate detection of live Salmonella spp. in food samples by viability PCR
Source: PLoS One. 2017 Dec 12;12(12):e0189302. doi: 10.1371/journal.pone.0189302 (PMC5726647; doi:10.1371/journal.pone.0189302)

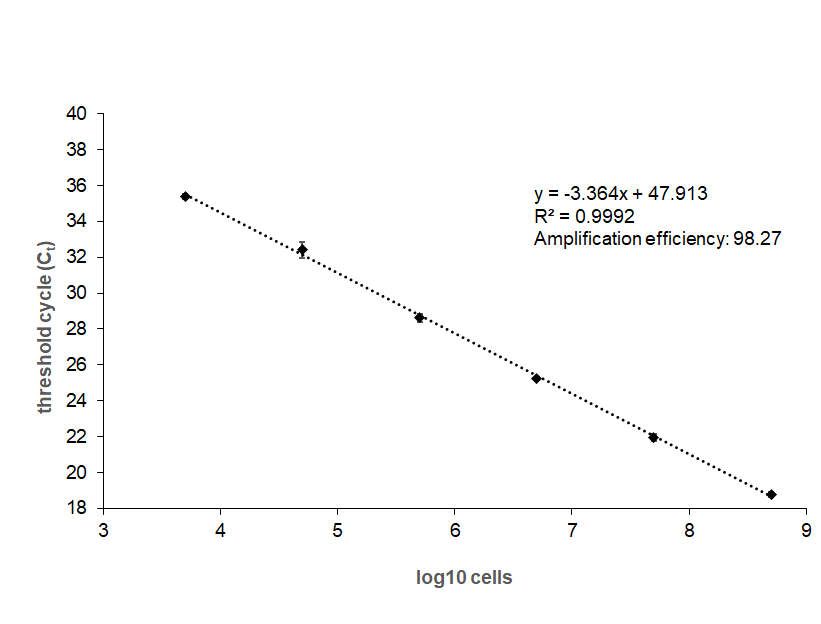

Supplement: S1 Fig — Cycle threshold (Ct) values were plotted against the corresponding log10 cell count (3.7–8.7). (TIF) [file pone.0189302.s002.tif]

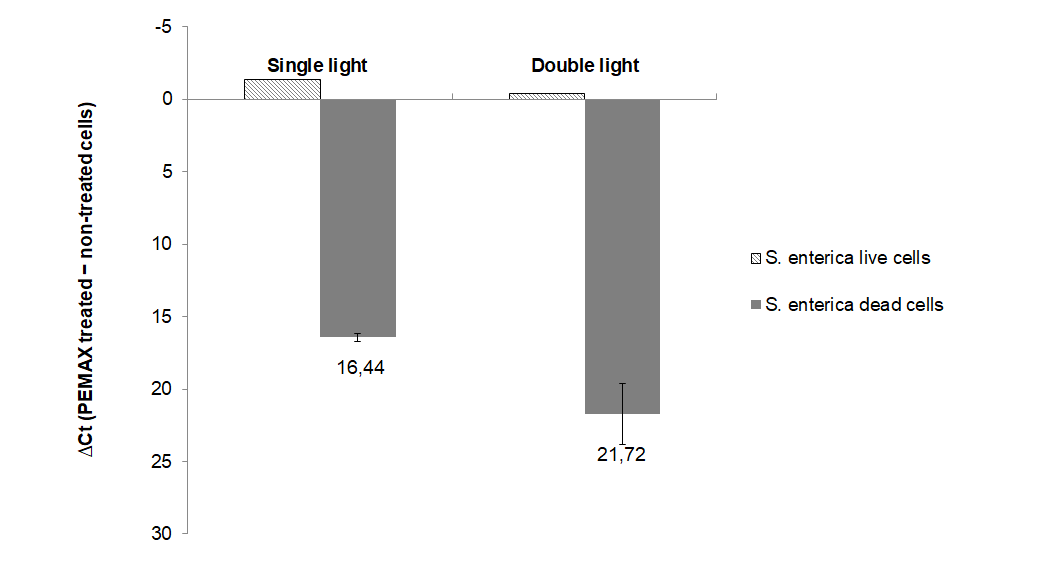

Supplement: S2 Fig — (TIF) [file pone.0189302.s003.tif]
